# Review of pharmacogenetics of antiseizure medications: focusing on genetic variants of mechanistic targets

**DOI:** 10.3389/fphar.2024.1411487

**Published:** 2024-08-20

**Authors:** Chih-Hsiang Lin, Chen-Jui Ho, Shih-Ying Chen, Yan-Ting Lu, Meng-Han Tsai

**Affiliations:** ^1^ Department of Neurology, Kaohsiung Chang Gung Memorial Hospital and Chang Gung University College of Medicine, Kaohsiung, Taiwan; ^2^ School of Medicine, College of Medicine, Chang Gung University, Taoyuan, Taiwan; ^3^ Department of Medical Research, Kaohsiung Chang Gung Memorial Hospital, College of Medicine, Chang Gung University, Kaohsiung, Taiwan

**Keywords:** antiseizure medication, drug-resistant epilepsy, genetic variants, pharmacogenetic studies, mechanistic targets

## Abstract

Antiseizure medications (ASMs) play a central role in seizure management, however, unpredictability in the response to treatment persists, even among patients with similar seizure manifestations and clinical backgrounds. An objective biomarker capable of reliably predicting the response to ASMs would profoundly impact epilepsy treatment. Presently, clinicians rely on a trial-and-error approach when selecting ASMs, a time-consuming process that can result in delays in receiving alternative non-pharmacological therapies such as a ketogenetic diet, epilepsy surgery, and neuromodulation therapies. Pharmacogenetic studies investigating the correlation between ASMs and genetic variants regarding their mechanistic targets offer promise in predicting the response to treatment. Sodium channel subunit genes have been extensively studied along with other ion channels and receptors as targets, however, the results have been conflicting, possibly due to methodological disparities including inconsistent definitions of drug response, variations in ASM combinations, and diversity of genetic variants/genes studied. Nonetheless, these studies underscore the potential effect of genetic variants on the mechanism of ASMs and consequently the prediction of treatment response. Recent advances in sequencing technology have led to the generation of large genetic datasets, which may be able to enhance the predictive accuracy of the response to ASMs.

## Introduction

Antiseizure medications (ASMs), previously called antiepileptic drugs (AEDs), are commonly used to manage seizures in individuals with epilepsy (McCorry et al., 2004). The ultimate goal of seizure treatment is to achieve seizure freedom, defined by the International League Against Epilepsy (ILAE) consensus as being free from seizures for a minimum of three times the longest preintervention inter-seizure interval or 12 months, whichever is longer (Kwan et al., 2010). To achieve this goal, physicians select ASMs based on factors such as age, gender, epilepsy syndrome, co-medications, and comorbidities. However, even an ASM carefully chosen by an experienced epileptologist can have a different response in different patients with similar epilepsy syndrome and/or underlying clinical status. Although more than 30 ASMs are currently available, approximately one-third of patients still have seizures after being treated with more than two ASMs (Kwan and Sander, 2004). Such patients are considered to have drug-resistant epilepsy (DRE), defined by the ILAE consensus as the failure of adequate trials of two tolerated and appropriately chosen ASMs to achieve sustained seizure freedom (Kwan et al., 2010). The effectiveness of controlling seizures or developing adverse drug reactions (ADRs) is unpredictable. Therefore, patients have to endure the consequences of inadequately controlled seizures or ADRs before DRE is confirmed, delaying the initiation of other management strategies to alleviate the seizures, such as a ketogenetic diet (Martin et al., 2016), epilepsy surgery (Rugg-Gunn et al., 2020), vagus nerve stimulation (Elliott et al., 2011), deep brain stimulation (Vetkas et al., 2022), or the use of cannabidiol (Gaston and Szaflarski, 2018). The use of ASMs relies on a trial-and-error approach, with careful adjustments of the dosage to achieve a balance between response and ADRs. This approach relies heavily on the clinicians’ expertise, and only a few objective biomarkers currently exist to aid in ASM selection. One notable success in this regard is the prediction of Stevens-Johnson syndrome/toxic epidermal necrolysis in Taiwanese and Southeast Asian populations harboring the human leucocyte antigen HLA-B*1502 allele (Hung et al., 2010). This discovery has influenced clinical practice, prompting genetic tests before prescribing relevant ASMs to prevent deadly skin reactions (Franciotta et al., 2009).

**FIGURE 1:**
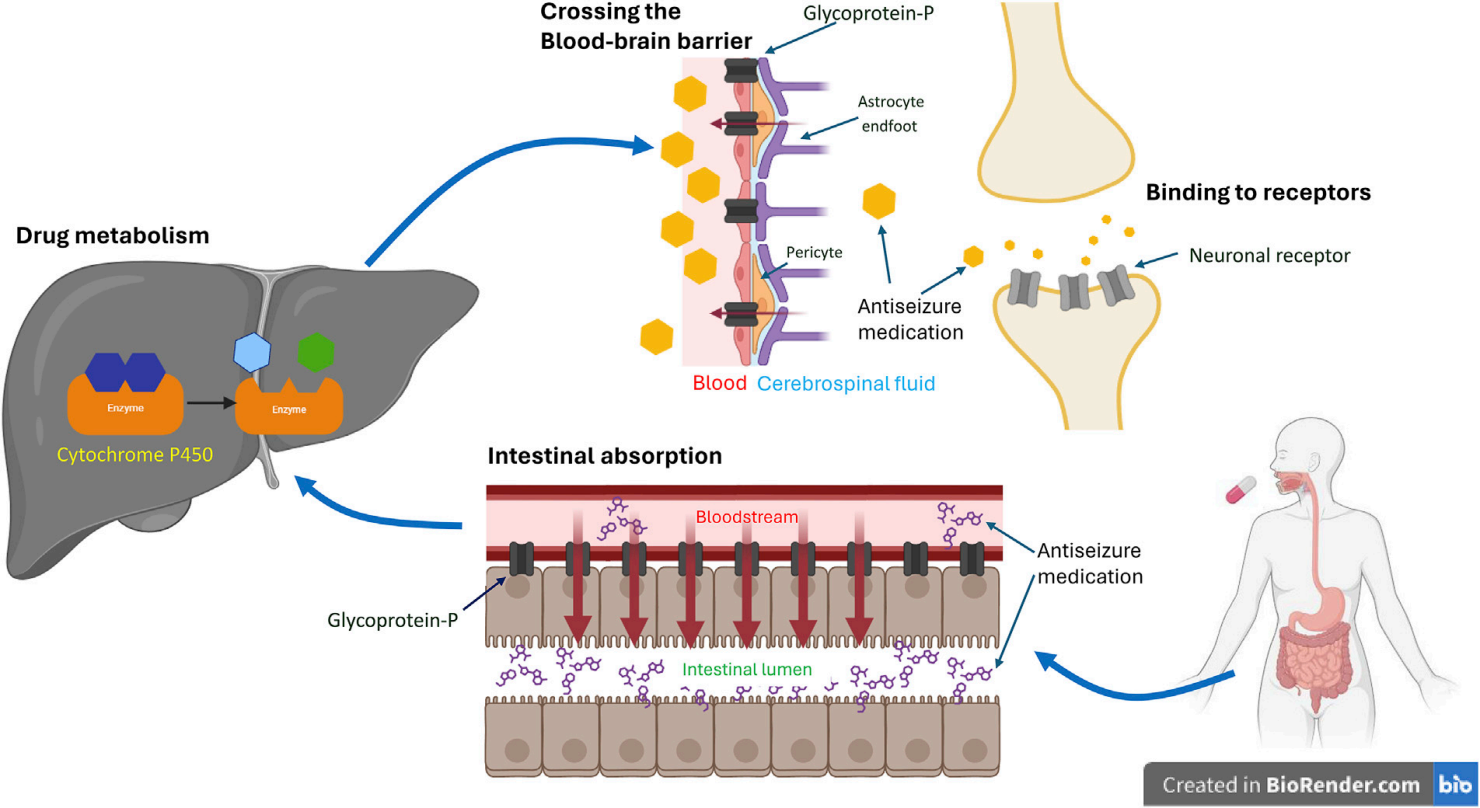
Coding Variants’ Impact on Antiseizure Medication. The schematic illustrates the impact of coding variants on the target, transportation, and metabolism of antiseizure medication (ASM). Typically, following oral administration of ASMs, the drug undergoes absorption in the gut, metabolism in the liver, and passage across the blood-brain barrier to reach its mechanistic target and exert its therapeutic effect. Genetic variants of efflux transporters in the gut, such as glycoprotein-P, can actively pump the drug back into the intestinal lumen, reducing its absorption. Similarly, variants of glycoprotein-P at the blood-brain barrier may facilitate the drug’s return to the bloodstream, decreasing its concentration in the brain. Genetic variants can also induce changes in metabolizing enzymes, such as cytochrome P450, in the liver. This can affect the rate of drug metabolism, leading to variable concentrations in the blood, differences in excretion, and variations in availability at its target site. Consequently, this may alter the effectiveness of ASMs. Additionally, genetic variants within the brain may modify neuronal receptors, compromising ASM’s ability to bind effectively and consequently diminishing its efficacy. *The figures were created with BioRender.com.

**FIGURE 2:**
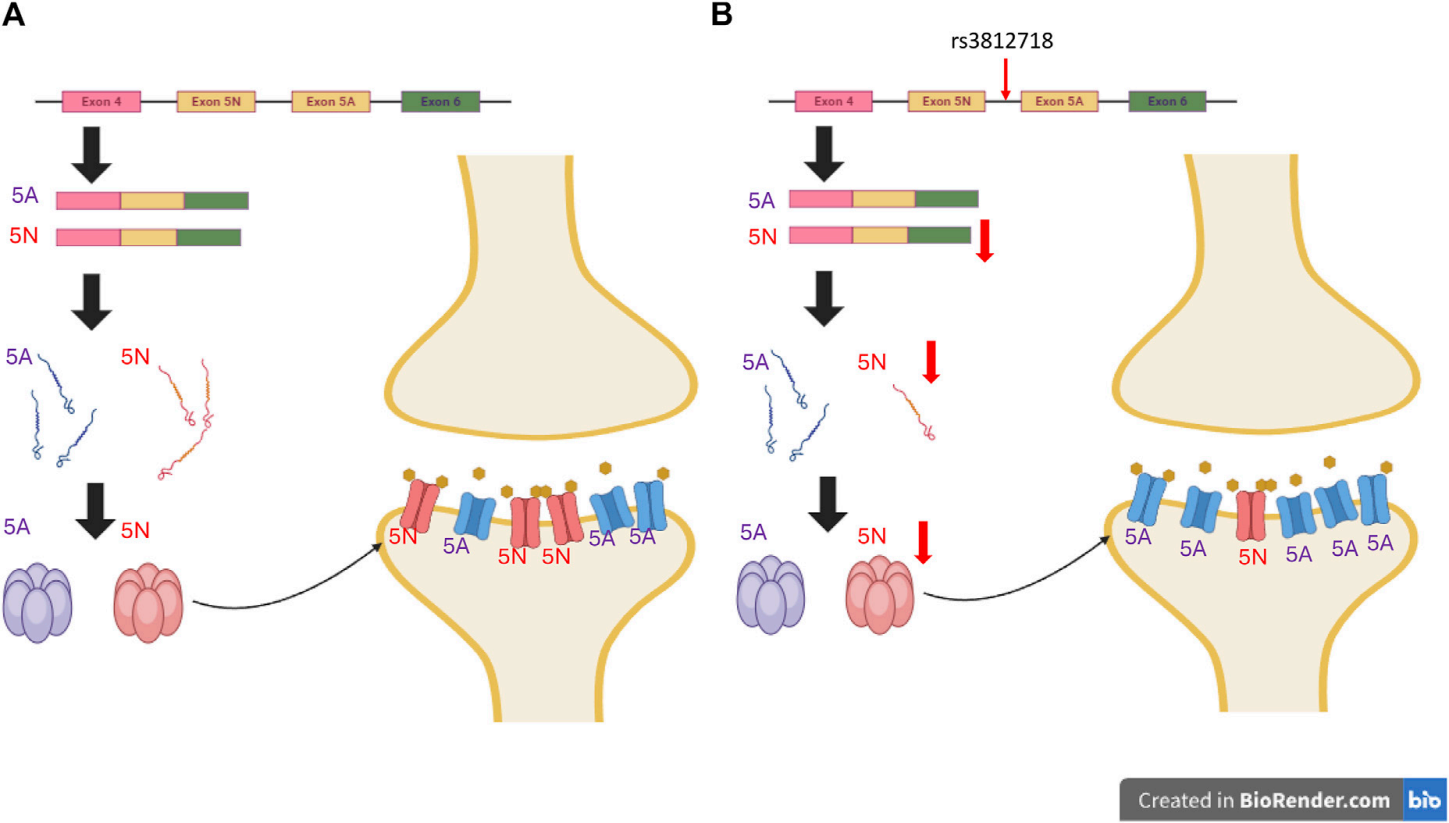
Impact of Non-Coding Genetic Variants on Antiseizure Medication Response. Genetic variants in non-coding regions, particularly introns, have the potential to disrupt typical splicing patterns. This disruption can lead to changes in protein expression levels and alter receptor functionality, consequently influencing responses to ASMs. The example provided in this Figure 2 focuses on SCN1A rs3812718. **(A)** The typical expression of *SCN1A* includes exon 5A and 5N. While 5N is more sensitive to sodium channel-blocking ASMs. **(B)** Conversely, within *SCN1A* carrying the rs3812718 variant, the expression of exon 5N is diminished, consequently reducing the abundance of receptors with heightened sensitivity to sodium channel-blocking ASMs. This decrease in receptors results in fewer channels being blocked, ultimately diminishing the antiseizure efficacy. (The ratio of 5A to 5N depicted in the figure does not accurately reflect the true conditions.). *The figures were created with BioRender.com.

ASMs exert their therapeutic effects by interacting with various targets in the central nervous system (CNS) (Das et al., 2012). Variations in genes encoding these targets theoretically have the potential to alter the response to ASMs. This can occur through changes in the coding region, resulting in amino acid sequence alterations that modify protein structures and their interaction with ASMs (Kimchi-Sarfaty et al., 2007) (Figure 1). Alternatively, variants in the non-coding region may interfere with protein synthesis, thereby affecting protein production and leading to varied responses to ASMs (Tate et al., 2005) (Figure 2). Pharmacogenetic studies offer a viable approach to predict the outcome of ASM treatment. However, despite extensive investigations, no biomarkers have yet demonstrated a predictive power comparable to that for dermatological side effects. This review focuses on pharmacogenetic studies of ASMs and their relevant mechanistic targets, and in particular on predicting the response to ASM treatment.

## Mechanistic targets of antiseizure medications

ASMs abolish aberrant neuronal excitability during epileptic discharge by altering the function of voltage-gated and ligand-gated ion channels (Meldrum and Rogawski, 2007). Voltage-gated ion channels are one of the main targets of ASMs including sodium channel (SCN) blockers, calcium current inhibitors, and potassium channel openers (Das et al., 2012). SCNs are responsible for the generation and propagation of action potential in neurons, and thus many ASMs act by reducing the high-frequency firing of voltage-dependent SCNs that occurs during a seizure (Abou-Khalil, 2016). Phenytoin (PHT), carbamazepine (CBZ), lamotrigine (LTG), oxcarbazepine (OXC), and lacosamide (LCM) are all SCN blockers (Brodie, 2017). Calcium channels contribute to the burst firing of neurons (Cain and Snutch, 2010) and control the release of presynaptic transmitters (Imbrici et al., 2004). Of the currently available ASMs, the main action of ethosuximide (Coulter et al., 1989), gabapentin (GBP) (Gee et al., 1996), and pregabalin (PGB) (Belliotti et al., 2005) is considered to be as blockers of calcium channels. The antiseizure effect of other ASMs may also in part be through the blockade of calcium channels, including PHT, phenobarbital, CBZ, OXC, zonisamide (ZNS), LTG, felbamate, topiramate (TPM), and valproate (VPA) (Abou-Khalil, 2019). Potassium channels are responsible for repolarizing the cell membrane and regulating the balance of excitability and resting of the neuron (Benarroch, 2009). Retigabine is currently the only ASM with the main action of enhancing resting membrane potential by opening potassium channels, however, it is not widely available due to discoloration of skin and conjunctiva (Gunthorpe et al., 2012). Other ASMs which may also modify potassium currents include ethosuximide (Leresche et al., 1998), LTG (Huang et al., 2004), and levetiracetam (LEV) (Madeja et al., 2003).

Several ligand-gated ion channels are also targets for ASMs, including gamma-aminobutyric acid (GABA) receptors and ionotropic glutamate receptors. GABA is the main fast inhibitory neurotransmitter, and it acts on GABA_A_ and GABA_B_ receptors in the CNS to terminate the bursting neuronal firing during seizures (Belelli et al., 2009). ASMs in this category, such as barbiturates and benzodiazepines can be used to treat status epilepticus (Czuczwar and Patsalos, 2001). Some ASMs modulate the disposition of GABA by inhibiting its catabolism, such as vigabatrin (Jung et al., 1977), or re-uptake, such as tiagabine (Krogsgaard-Larsen et al., 1987). Ionotropic glutamate receptors consist of three major forms, namely α-amino-3-hydroxy-5-methyl- 4-isoxazole propionic acid (AMPA), N-methyl-D-aspartate (NMDA), and kainate receptors, all of which they mediate the excitatory neurotransmission of glutamate (Reiner and Levitz, 2018). Perampanel (PER), a non-competitive AMPA receptor antagonist (Plosker, 2012), is currently the only ASM in this category.

Carbonic anhydrase (CA) regulates the flux of bicarbonate to maintain ion and pH homeostasis in neurons (Krishnamurthy et al., 2008). Inhibition of CA has been postulated to contribute to some of the antiseizure effects of acetazolamide, TPM, and ZNS (Das et al., 2012). The exact mechanism of how these CA inhibitors halt seizures is still unknown and no pharmacogenetic studies of variants in CA genes have been conducted to date.

LEV and brivaracetam share the same target, synaptic vesicle protein 2A (SV2A) (Rossi et al., 2022), and they exert their antiseizure effects by regulating exocytosis from synaptic vesicles and inhibiting the release of presynaptic neurotransmitters.

There are also broad-spectrum ASMs with multiple targets, such as ZNS (SCN blockade, calcium blocker, and CA inhibitor) (Brodie et al., 2012) and TPM (SCN blockade, GABA enhancer, CA inhibitor, and AMPA blockade) (Gryder and Rogawski, 2003), and ASMs with still unclear mechanisms, such as VPA (Romoli et al., 2019). Even for ASMs with known major targets, additional antiseizure mechanisms could arise from interactions with other minor targets (Sills and Rogawski, 2020). As a result, genetic variations of multiple receptors could result in the varied responses to ASMs, especially in patients taking polytherapy for seizure control.

With advances in the understanding of epileptogenesis, researchers are exploring innovative medications that target the pathogenic pathways implicated in seizure development or progression, beyond ion channels. These promising treatments show potential in effectively controlling seizures, and two are either undergoing human studies or are already in use. Cholesterol is metabolized to 24-hydroxycholesterol in the CNS by the cholesterol 24-hydroxylase (CH24H) enzyme (Popiolek et al., 2020). Lowering the cholesterol level in glial cells can result in an increased extracellular glutamate level (Tian et al., 2010) that may contribute to the genesis of seizures (Dejakaisaya et al., 2021). Soticlestat (TAK-935), a selective inhibitor of CH24H, demonstrated efficacy in decreasing seizure frequency among patients with Dravet syndrome during a phase 2 clinical trial (Hahn et al., 2022). Another example is the inhibition of mammalian target of rapamycin (mTOR). The mTOR pathway participates in cellular signaling and plays a crucial role in neuronal development (Lipton and Sahin, 2014). Tuberous sclerosis complex and focal cortical dysplasia are associated with hyperactivation of the mTOR pathway (Crino, 2015). Everolimus, a TOR inhibitor, can reduce seizure frequency in patients with tuberous sclerosis complex (Samueli et al., 2016). Numerous compounds are currently undergoing rigorous cellular and animal studies. For an in-depth review of the latest advances in epilepsy treatment targeting novel pathways, refer to Belete (2023).

Advances in computational bioinformatics have opened a new avenue for discovering novel targets for ASMs. In a recent study, data from genome-wide association studies for epilepsy were combined with data from proteome-wide association studies, transcriptome-wide association studies, and chemical-related gene set enrichment analysis, with the aim of identifying promising new targets for seizure treatment (Lu et al., 2023). Five genes were found to be significantly associated with epilepsy, and consequently may be new candidate epilepsy genes. This study showcased the potential of using bioinformatics techniques to identify new mechanisms potentially relevant to biological processes.

## Association studies of genetic variants among mechanistic targets

We conducted a systematic search of PubMed to find relevant articles regarding the response to ASMs and the mechanistic targets previously discussed. We also included data from our recent studies, which not only enhanced the breadth of the review but also contributed original insights to enhance the overall understanding of the subject. Initially, we searched for keywords including “genetic polymorphisms” and “drug resistance epilepsy” in studies published from 2005 to 2023. Studies from any geographic location and including subjects of any ethnicity, gender, and age were included. Case reports were excluded from this review. Non-English articles or abstracts without full text were also excluded. Titles and abstracts of the articles retrieved from the initial search were screened for relevance, and full texts of potentially relevant articles were reviewed. We also extracted and examined the reference lists of these articles for additional relevant papers based on their titles and abstracts. We then searched for studies involving the mechanistic targets of ASMs, including sodium channels, calcium channels, potassium channels, GABA receptors, and ionotropic glutamate receptors. Subsequently, all available full-text articles were evaluated, and those not relevant to the response to ASMs or the mechanistic targets were excluded. The collected data were synthesized and compared to provide an overview of the progress in ASM pharmacogenetics, including conclusions and discussions regarding the limitations and future prospects.

The search returned 223 results. Thirty-five articles were excluded because they were either not relevant to the response to ASMs, focused on ribonucleic acid rather than genes, or were not human studies. The remaining articles contained 38 reviews or meta-analysis articles and 150 articles related to genetic polymorphisms and drug response. After reviewing these 188 articles and their references, 26 were selected, with 13 related to SCNs, two to calcium channels, four to potassium channels, five to GABA receptors, one to synaptic vesicle proteins, and one to ionotropic glutamate receptors. The remaining articles that mentioned the association between drug response and transporter or metabolic genes, although not included in the scope of our review, are also briefly discussed in this article.

## Sodium channel gene variations

Since the first introduction of PHT in 1936 (Merritt and Putnam, 1984), SCN blockers have been one of the most popular treatments for both focal and generalized seizures. As a result, the relationship between SCN gene variants and response to ASMs has received much attention.

Most previous studies have focused on single-nucleotide polymorphisms (SNPs) of SCN genes and the dose of SCN blockers. The intronic *SCN1A* rs3812718 variant is one of the most extensively studied variants. In 2005, Tate et al. (2005) reported that this variant was associated with the maximum dose of PHT and CBZ required to control seizures in European patients. rs3812718 is located between exon 5 and 6 of the *SCN1A* gene and affects the splicing of exon 5, which results in decreasing the percentage of transcripts containing exon 5N (neonatal form) of *SCN1A* (Copley, 2004) (Figure 2). By decreasing the amount of the neonatal form of *SCN1A*, which is more sensitive to SCN blockers (Thompson et al., 2011), the authors suggested that patients with this SNP may need a higher dose or become more resistant to SCN blockers. This association was replicated in one Taiwanese study, where rs3812718 was also associated with a higher maintenance dose of CBZ monotherapy (Hung et al., 2012).

Later reports focused on the association between SNPs in SCN genes and DRE instead of the dose of ASMs. One report regarding Japanese patients with epilepsy found that *SCN1A* rs3812718 was associated with resistance to CBZ but not the dose of CBZ (Abe et al., 2008). In Chinese patients receiving CBZ or OXC, *SCN1A* rs3812718 was associated with DRE (Ma et al., 2014). In addition, a Greek study found that *SCN1A* rs3812718 was associated with resistance to SCN blocker monotherapy (PHT, CBZ, OXC, or LTG) and increased dosing of SCN blockers in patients responsive to SCN blockers (Angelopoulou et al., 2017), however, they did not find an association between *SCN1A* rs3812718 and the responsive to ASM polytherapy that contained SCN blockers. Other studies have reported conflicting results about the effect of *SCN1A* rs3812718 on the responsive to SCN blockers. An Italian study found that *SCN1A* rs3812718 was not associated with the responsive to CBZ/OXC, the dose of CBZ/OXC, or DRE (Manna et al., 2011). In the study, DRE was defined as having at least one seizure per month within 2 years of follow-up under three or more ASMs. A study from northern India also reported that *SCN1A* rs3812718 was not associated with the response to CBZ/OXC or DRE (Kumari et al., 2013), which they defined as at least four seizures over 1 year under three or more ASMs. In addition, a Spanish study did not find an association between *SCN1A* rs3812718 and DRE, defined as having at least four seizures under more than three ASMs 1 year before recruitment (Sanchez et al., 2010). However, this study did not specifically focus on the use of SCN blockers.

Besides *SCN1A* rs3812718, other SNPs of SCN receptor genes have also been associated with drug responsiveness. *SCN2A* rs17183814 was associated with DRE in Indian patients, defined as at least four seizures in 1 year under three or more ASMs (Lakhan et al., 2009), and an Australian study found that SCN1A rs10188577 was moderately associated with DRE, albeit without statistical significance (Yip et al., 2014).

These studies generally focused on less than or around a dozen SNPs, however, more genetic variants in SCNs are likely to contribute to the response to SCN-blocking ASMs. A study on Hong Kong Chinese patients investigated 27 SNPs in *SCN1A*, *SCN2A*, and *SCN3A* genes, and found that *SCN2A* rs2304016 was associated with both DRE, defined as one or more seizures per month for 1 year under ASM treatment, and resistance to SCN blockers, defined as patients with DRE when their most recent ASM regimen consisted of SCN-blocking ASMs (Kwan et al., 2008). Another cohort study evaluated 39 polymorphisms in the *SCN1A*, *SCN2A*, and *SCN3A* genes from patients in Malaysia and Hong Kong, and found no association between gene polymorphisms and responsive to CBZ or VPA monotherapy (Haerian et al., 2013). One study involving Taiwanese patients identified rs55742440 in *SCN1B*, among 21 coding SNPs in *SCN1A*, *SCN1B*, *SCN2A*, and *SCN9A*, as being associated with resistance to SCN blockers (Lin et al., 2021). This resistance was defined as the failure of the most recently administered SCN blocker to achieve seizure freedom for at least 1 year.

The methodologies in these studies were heterogeneous (Table 1), including the definition of DRE, the specific ASM under investigation, and the inclusion of different genetic variants. These differences make it challenging to compare the results and provide meaningful clinical predictions. However, most of these studies do imply that genetic variants in the SCN genes have an impact on the responsive to SCN-blocking ASMs.

**TABLE 1:** Studies focusing on the genetic variants of the sodium channel genes.

Author date.	Genes studied	Ethnicity and number of patients	Result of ASM use	Observation
Tate et al. (2005)	*SCN1A* rs590478 *SCN1A* rs8191987 *SCN1A* rs3812718 *SCN1A* rs2126152 *ABCB1* rs1045642 *CYP2C9* rs1799853 *CYP2C9* rs1057910	European patients with 448 patients using PHT and 425 patients using CBZ	Maximum dose of CBZ and PHT taken by the patients.	*SCN1A* rs3812718 is associated with the maximum dose of CBZ and PHT. The CYP2C9*3 allele is associated maximum dose of PHT.
Abe et al. (2008)	*SCN1A* rs3812718	228 Japanese patients	CBZ-responsive epilepsy was defined as not experiencing seizures of any type for a minimum of 1 year under a stable dose of CBZ. DRE is defined as uncontrolled seizures over a year with three or more different ASMs.	*SCN1A* rs3812718 is associated with CBZ-resistant epilepsy but not the maximum or maintenance dose of CBZ.
Kwan et al. (2008)	27 SNPs in *SCN1A*, *SCN2A*, and *SCN3A*.	272 Chinese patients	Enrolled patients taking ASMs of any type. DRE is defined as one seizure or more per month within 12 months, despite treatment with two or more ASMs.	*SCN2A* rs2304016 is associated with DRE
Lakhan et al. (2009)	*SCN1A* rs2298771 *SCN2A* rs17183814	336 North Indian patients	Enrolled patients taking ASMs of any type. DRE is defined as the occurrence of at least four seizures for 1 year with three appropriate ASMs at maximum tolerated doses	*SCN2A* rs17183814 is associated with DRE.
Sanchez et al. (2010)	*SCN1A* rs2298771 *SCN1A* rs3812718 *CYP2C9* rs1799853 *CYP2C9* rs1057910 *CYP2C19* rs4244285 *CYP2C19* rs4986893 *UGT1A1* rs35350960 *UGT2B7* rs28365063 *UGT2B7* rs7668258 *ABCB1* rs1045642 *ABCB1* rs2032582	289 Caucasian patients	Enrolled patients taking ASMs of all types. DRE is defined as the occurrence of at least four seizures over the year with trials of more than three appropriate ASMs at maximal tolerated doses.	*SCN1A* polymorphisms are not associated with DRE. *ABCB1* rs1045642 and *ABCB1* rs2032582 are associated with DRE.
Manna et al. (2011)	*SCN1A* rs3812718	883 Italian patients	Enrolled patients taking CBZ or OXC. DRE is defined as the persistence of seizures during the previous 2 years with a frequency of at least one seizure/month, despite current or previous treatment with three or more appropriate ASMs, either alone or in combination, and at the highest tolerated dose.	No association between *SCN1A* rs3812718 and DRE.
Hung et al. (2012)	*SCN1A* rs3812718 *SCN1A* rs2298771 *SCN2A* rs17183814 *EPHX1* rs1051740 *EPHX1* rs2234922 *UGT2B7* rs7668258 *UGT2B7* rs7438135 *UGT2B7* rs28365062 *UGT2B7* rs7439366 *ABCB1* rs1128503 *ABCB1* rs2032582 *ABCB1* rs1045642 *ABCC2* rs717620 *ABCC2* rs2273697	234 Taiwanese patients	Enrolled patients taking CBZ monotherapy with seizure free for 1 year.	*SCN1A* rs3812718 and *EPHX1* rs1051740 are associated with higher doses of CBZ.
Kumari et al. (2013)	*SCN1A* rs3812718	Patients from north India. 231 patients using CBZ or OXC monotherapy. 272 patients using multiple ASMs.	Two patient groups include CBZ/OXZ monotherapy and a combination of various ASMs. DRE is defined as the occurrence of at least four seizures for 1 year with three or more appropriate ASMs.	*SCN1A* rs3812718 was not associated with drug responsiveness either in CBZ/OXZ treated monotherapy or multi-drug polytherapy
Haerian et al. (2013)	39 SNPs in the *SCN1A*, *SCN2A*, and *SCN3A*.	1,504 patients from Hong Kong and Malaysia	DRE is defined as the occurrence of seizures at any time during 1 year while undergoing treatment with CBZ or VPA monotherapy at maximally tolerated therapeutic dosages.	None of the polymorphisms in *SCN1A*, *SCN2A*, and *SCN3A* are associated with DRE
Ma et al. (2014)	*SCN1A* rs2298771 *SCN1A* rs3812718 *SCN2A* rs17183814 *SCN2A* rs2304016 *ABCC2* rs3740066 *ABCC2* rs2273697	453 Chinese patients.	All patients received CBZ or OXC (either monotherapy or polytherapy). DRE is defined as the failure of adequate trials following two tolerated, appropriately chosen, and administered ASM schedules (whether as monotherapies or in combination) to achieve seizure-free for 1 year.	*SCN1A* rs3812718 and *ABCC2* rs2273697 are associated with CBZ/OXC-resistant epilepsy.
Yip et al. (2014)	*SCN1A* rs1813502 *SCN1A* rs1461195 *SCN1A* rs1020853 *SCN1A* rs6432860 *SCN1A* rs1972445 *SCN1A* rs10188577 *SCN1A* rs7607543 *SCN1A* rs11686142 *SCN1A* rs1461197	519 Caucasian patients	Enrolled patients taking PHT, CBZ, OXC, LTG, VPA, and TPM DRE is defined as having at least four seizures over the year before recruitment with trials of two or more SCN-blocking ASMs at maximal tolerated doses.	*SCN1A* rs10188577 has moderate association with DRE.
Angelopoulou et al. (2017)	*SCN1A* rs3812718	200 Italian patients	Patients taking monotherapy with PHT, CBZ, OXC, or LTG. The paper mentioned the definitions of drug responsiveness and drug resistance are those proposed by the Task Force of the ILAE Commission on Therapeutic Strategies (2009) without further description.	*SCN1A* rs3812718 is associated with monotherapy-resistant epilepsy and with maximum doses of ASMs effectiveness in monotherapy.
Lin et al. (2021)	21 SNPs in *SCN1A*, *SCN1B*, *SCN2A*, and *SCN9A*	200 Taiwanese patients	Enrolled patients taking various ASMs. DRE is defined as not achieving seizure freedom lasting for ≥12 months after taking the last SCN-blocking ASM.	rs55742440 in *SCN1B* is associated with DRE.

Abbreviations: ASM, antiseizure medication; CBZ, carbamazepine; DRE, drug-resistant epilepsy; LTG, lamotrigine; OXC, oxcarbazepine; PHT, phenytoin; SCN, sodium channel; SNP, single-nucleotide polymorphisms; TPM, topiramate; VPA, valproic acid.

## Calcium channel gene variations

A Chinese study focusing on the association of DRE and with polymorphisms of calcium channel genes, including 15 SNPs in *CACNA1A*, *CACNA1C*, and *CACNA1H*, found that no SNPs but the TAGAA haplotype of *CACNA1A* was associated with DRE (Lv et al., 2015). Another study involving Jordanian patients found that *CACNG5* rs740805 and *GABRA1* rs2279020 were associated with DRE (Al-Eitan et al., 2020). Both of these studies included patients taking various combinations of ASMs, although many ASMs can inhibit calcium channels (Abou-Khalil, 2019). These studies only suggested a relationship between genetic variants of calcium channels and DRE.

## Potassium channel gene variations

A Chinese study identified that *KCNJ10* rs12402969, among seven other SNPs in *KCNJ10*, was associated with DRE, defined as experiencing four seizures under at least three ASMs during the previous year (Guo et al., 2015). However, another Chinese study, using the same DRE definition found no association between eight SNPs in *KCNA1*, *KCNA2*, and *KCNV2* with DRE (Qu et al., 2017). Similarly, a separate Chinese study reported no association between nine SNPs in *KCNJ10* and DRE (Zhu et al., 2020). In addition, a Jordanian-Arab study focusing on patients initiating treatment with VPA and CBZ as their first ASM revealed no association between seven SNPs in *KCNA1*, *KCNA2*, and *KCNV2* and the response to these ASMs (Al-Eitan et al., 2018). Notably, while *KCNJ10* rs12402969 was included in the studies by Guo et al. and Zhu et al., it was not considered in the studies by Qu et al. and Al-Eitan et al., leaving the association between this SNP and DRE inconclusive.

With various ASMs included, these studies suggest that genetic variants in potassium channels may have a minimal, if any, effect on DRE. This may not be surprising given that most ASMs do not target potassium channels. Therefore, associations between the response to potassium channel-targeted ASMs and genetic variants in potassium channel genes remain to be fully elucidated. Potassium channel enhancer represents a potential avenue for future ASM development.

## Gamma-aminobutyric acid receptor gene variations

The *GABRG2* rs211037 variant has been extensively studied across various ethnicities. In an Egyptian study, *GABRG2* rs211037 was linked to DRE, defined as the inability to achieve 1 year of seizure freedom while using two ASMs (Abou El Ella et al., 2018). In Chinese patients, resistance to VPA, defined as having seizures with at least 12 months of VPA use, was associated with the heterozygous CT genotype of the *GABRG2* rs211037 polymorphism (Lu et al., 2021). In addition, an Indian study found that *GABRA1* rs2279020 but not *GABRG2* rs211037 was associated with DRE, defined as at least four seizures within 1 year under three ASMs (Kumari et al., 2010). However, a Pakistan study failed to find an association between *GABRG2* rs211037 and DRE, defined as no change in seizure frequency under two ASMs (Saleem et al., 2022). Another study from southern India that included four GABA_A_ receptor subunit SNPs also found no association with DRE, defined as having a seizure frequency of 12 per year under at least two ASMs for at least 2 years (Balan et al., 2013). In contrast, a meta-analysis showed that *GABRG2* rs211037 was related to DRE in the Asian population (Hu et al., 2023).

These studies did not specifically analyze the types of ASMs taken, except for one study which was limited to VPA, a medication known to enhance the effect of GABA (Loscher, 2002). GABA-enhancing ASMs such as barbiturates and benzodiazepines are predominantly used in patients with status epilepticus (Czuczwar and Patsalos, 2001). Nonetheless, there is currently a lack of studies specifically addressing this aspect.

## Synaptic vesicle protein gene variations

A European study evaluated 86 common variants in *SV2A*, *SV2B*, and *SV2C,* but found no associations between these variants and the response to LEV (Lynch et al., 2009). Another study investigated genetic susceptibility to the neuropsychiatric side effects of LEV, and despite conducting genome-wide association analysis and rare-variant analysis, no significant associations were identified (Campbell et al., 2022). However, intriguingly, this study revealed that patients experiencing neuropsychiatric side effects had an elevated polygenic risk score for schizophrenia (Campbell et al., 2022). Taken together, these findings suggest that neither the response to LEV nor the occurrence of psychiatric adverse effects was linked to variability in synaptic vesicle genes, but rather that it was influenced by underlying genetic predispositions.

## Ionotropic glutamate receptors

PER, an AMPA receptor antagonist, is currently the only ASM in this category. Lin et al. investigated the association between rare coding genetic variants in glutamate receptor genes and ADR, as well as responsive to PER, in Taiwanese patients undergoing PER treatment (Lin et al., 2023). Resistance to PER was defined as not achieving seizure freedom for 1 year under PER treatment. Although Lin et al. did not discover a direct association between individual genetic variants and drug response, they observed that enrichment of genetic variants within the glutamate receptor gene group was statistically associated with the occurrence of ADRs. Upon further analysis, they found that rare variant enrichment in the glutamate ionotropic receptor delta subunit was nominally associated with ADR. In addition, gene burden analysis revealed a nominal association between *GRID1* and ADRs. These findings suggest that studies focusing on genetic variants in mechanistic targets hold the potential not only to predict responsiveness but also to predict the occurrence of ADRs.

In summary, genetic variants in ASM targets beyond SCN (as outlined in Table 2) present conflicting findings. Many of the previous investigations did not specifically target the mechanistic action of ASMs, making it challenging to ascertain whether the response to ASMs was correlated with genetic variants in their mechanistic targets. As relatively little research has investigated the correlation between the response to LEV or PER and their respective targets, further investigations are warranted.

**TABLE 2:** Studies focus on genetic variants other than the sodium channel genes.

Author date.	Gene studied	Ethnicity and number of patients	Result of ASM use	Observation
Calcium channel				
Lv et al. (2015)	*CACNA1A* rs2074880 *CACNA1A* rs10416717 *CACNA1A* rs7254351 *CACNA1A* rs16030 *CACNA1A* rs2248069 *CACNA1C* rs2239128 *CACNA1C* rs215976 *CACNA1C* rs7316246 *CACNA1C* rs216008 *CACNA1C* rs12813888 *CACNA1H* rs1054645 *CACNA1H* rs3794619 *CACNA1H* rs7191246 *CACNA1H* rs11640796 *CACNA1H* rs3751664	480 Chinese patients	Enrolled patients taking various ASMs. DRE is defined as failure to achieve seizure-free for at least three times the longest inter-seizure interval before starting a new intervention and not less than 12 months after an adequate trial of two appropriate tolerated ASMs.	No SNPs are associated with DRE but the TAGAA haplotype of *CACNA1A* was associated with DRE.
Al-Eitan et al. (2020)	*CACNG5* rs740805 *GABRG2* rs209337 *GABRA1* rs2279020 *SLC1A1* rs10815018 *SLC6A1* rs10510403 *FAM131B* rs4236482 *GPLD1* rs1126617 *GPLD1* rs2076317 *F2* rs1799963	296 Jordanian patients	Enrolled patients taking various ASMs. DRE is defined as failure to achieve seizure-free for at least three times the longest inter-seizure interval before starting a new intervention and not less than 12 months after an adequate trial of two appropriate tolerated ASMs.	*CACNG5* rs740805, *FAM131B* rs4236482, and *GABRA1* rs2279020 are associated with DRE.
Potassium channel				
Guo et al. (2015)	*KCNJ10* rs1053074 *KCNJ10* rs1130183 *KCNJ10* rs12729701 *KCNJ10* rs12402969 *KCNJ10* rs1186688 *KCNJ10* rs1186685 *KCNJ10* rs6690889 *KCNJ10* rs1890532	483 Chinese patients	Enrolled patients taking various ASMs. DRE is defined as having at least four seizures during the previous year with at least three ASMs at maximal tolerated doses.	*KCNJ10* rs12402969 is associated with DRE.
Qu et al. (2017)	*KCNA1* rs2227910 *KCNA1* rs112561866 *KCNA1* rs7974459 *KCNA2* rs3887820 *KCNV2* rs7029012 *KCNV2* rs10967705 *KCNV2* rs13285989 *KCNV2* rs10967728″	483 Chinese patients	DRE is defined as at least four seizures during the previous year while trying at least three ASMs at maximally tolerated doses.	No association between DRE and the studied SNPs.
Al-Eitan et al. (2018)	*KCNA1* rs2227910 *KCNA1* rs112561866 *KCNA1* rs7974459 *KCNA2* rs3887820 *KCNV2* rs7029012 *KCNV2* rs10967705 *KCNV2* rs10967728	296 Jordanian-Arab patients	Enrolled patients taking VPA for generalized epilepsy and CBZ for focal epilepsy as the first ASM. Good responder was defined as patients who require the lowest ASM doses or have taken only one drug without relapse in the past 6 months. Poor responders are patients requiring the highest ASM doses or who have taken more than one drug.	No SNPs are associated with the response of ASMs.
Zhu et al. (2020)	*KCNJ10* rs12122979 *KCNJ10* rs1186685 *KCNJ10* rs6690889 *KCNJ10* rs2486253 *KCNJ10* rs1186675 *KCNJ10* rs12402969, *KCNJ10* rs12729701 *KCNJ10* rs1890532 *KCNJ10* rs3795339 *AQP4* rs1058424 *AQP4* rs3763043 *AQP4* rs35931	510 Chinese patients	Enrolled patients taking various ASMs. DRE is defined as at least four seizures during the previous year while trying at least two appropriate chosen ASMs.	No SNPs are associated with the response of ASMs.
Gamma-Aminobutyric Acid Receptor				
Kumari et al. (2010)	*GABRA1* rs2279020 *GABRG2* rs211037	395 North Indian patients	Enrolled patients taking various ASMs. DRE is defined as the occurrence of at least four seizures for 1 year with three appropriate ASMs at maximum tolerated doses.	*GABRA1* rs2279020 is associated with DRE but not *GABRG2* rs211037
Balan et al. (2013)	*GABRA1* rs2279020 *GABRB2* rs2229944 *GABRB2* rs211037 *GABRA6* rs3219151	441 South Indian patients	Enrolled patients taking various ASMs. DRE is defined as unresponsive to at least two monotherapy trials and one duo therapy trial, each of at least 6 months duration, and had seizure frequency of at least 12 per year for at least 2 years.	None of the SNPs are associated with DRE
Abou El Ella et al. (2018)	*GABARG2* rs211037	100 Egyptian patients	Enrolled patients taking various ASMs. DRE is defined as failure to achieve sustained seizure-free for 1 year under two tolerated and appropriately chosen ASMs.	*GABARG2* rs211037 is associated with DRE
Lu et al. (2021)	*GABRG2* rs211037	96 Chinese patients	Enrolled patients taking VPA alone or in combination with other ASMs for at least 12 months. Not responding to VAP is defined as recurrent seizure with a sufficient dose of VPA achieving therapeutic blood concentration (50–100 μg/mL).	*GABRG2* rs211037 was associated with recurrent seizures under VPA use.
Saleem et al. (2022)	*GABRG2* rs211037	142 Pakistani patients	Enrolled patients taking various ASMs. DRE is defined as consistent seizure frequency despite treatment with a maximum tolerated dose of two established ASMs for at least a couple of years	Only *GABRG2* rs211037 is associated with DRE.
Synaptic vesicle protein				
Lynch et al. (2009)	Eighty-six SNPs in *SV2A*, *SV2B*, and *SV2C*	247 patients from the United Kingdom and 290 Irish patients	Enrolled patient using LEV after failure to respond to at least two ASMs. Not responding to LEV is defined as seizure-free for at least 6 months under LEV use.	No SNPs are associated with the response of LEV.
Ionotropic glutamate receptors				
Lin et al. (2023)	Rare variants in the 26 glutamate receptor genes, *GRIA1-4*, *GRID1-2*, *GRIK1-5*, *GRIN1, GRIN2A-D*, *GRIN3A-B*, and *GRM1-8*	83 Taiwanese patients	Enrolled patients received PER in the regiment of their ASMs. Resistance to PER is defined as failure to achieve seizure-free for a least 1 year while the PER was the patient’s last ASM.	None of the rare genetic variants in the glutamate receptor genes are associated with responsiveness to PER.

Abbreviations: ASM, antiseizure medication; CBZ, carbamazepine; DRE, drug-resistant epilepsy; SNP, single-nucleotide polymorphisms; LEV, levetiracetam; PER, perampanel; VPA, valproic acid.

## Other factors related to drug-resistant epilepsy

Besides the mechanistic targets of ASMs, other factors also contribute to DRE, including alterations in the drug delivery system across the brain, and pharmacodynamic modifications that change the absorption or metabolism of ASMs (Loscher et al., 2020). In addition, Mendelian genetic factors may also determine the disease severity and increase refractoriness to ASM treatment, such as developmental epileptic encephalopathy.

Efflux transporters (Figure 1) in the endothelial cells of the blood-brain barrier (BBB) can hamper the ability of ASMs to enter the CNS and decrease their concentration in epileptogenic tissues (Kwan and Brodie, 2005). The ATP binding cassette (ABC) transporter superfamily is the major efflux transporter on the BBB that limits the access of ASMs to the target sites (Qosa et al., 2015). Glycoprotein-P (P-gp), one of these efflux transporters, has been shown to actively remove ASMs from the BBB back into the bloodstream (Miller et al., 2008). Moreover, it has been shown to be over-expressed in epileptogenic foci of the brain (Marchi et al., 2004), further preventing the seizure-suppression effect of ASMs. Studies on the genetic polymorphism of ABC transporter genes and the relationship between the response to ASMs are conflicting (Seo et al., 2006; Ozgon et al., 2008; Meng et al., 2011). For a comprehensive review of the impact of genetic polymorphisms of ABC transporter and the responsive to ASMs, see Leandro et al. (2019).

Drug-metabolizing genes are polymorphic and can influence the biotransformation of many drugs (Meyer and Zanger, 1997). These polymorphisms may result in a higher plasma drug level under standard dosage that leads to the occurrence of ADRs or increases enzyme activity to make the ASMs less effective (Eadie, 1991) (Figure 1). Drug metabolism occurs in two phases. Phase 1 reactions involve the addition of a functional group, mostly hydroxylation, by the cytochrome P450 (CYP) family followed by a phase 2 metabolism of various conjugating reactions that increase hydrophilicity and facilitate renal excretion of the drug. The enzymes included in this process are CYP, UDP-glucuronyl transferase (UGT), epoxide hydroxylase (EPHX), glutathione S-transferase (GST), microsomal GST, N-acetyltransferase (NAT), and sulfatase (SULF) (Ferraro and Buono, 2005). Various studies including polymorphisms in CYP (van der Weide et al., 2001; Lopez-Garcia et al., 2017; Makowska et al., 2021), EPHX (Hung et al., 2012), and UGT (Ma et al., 2015) have been conducted, with conflicting results.

Recently, the EpiPGX Consortium investigated the complex genetic factors associated with responsive to ASMs (Wolking et al., 2020). The study included 1,622 patients and 808,583 genetic variants, which is by far the largest cohort of pharmacogenetic studies regarding ASM. They enrolled patients taking LEV, VPA, and LTG for seizure control and studied rare genetic variants presumed to have functional consequences. A non-presupposed gene-based enrichment analysis did not find associations between any of the coding variants and response to ASM. The gene-set-based enrichment analysis, focusing on ASM target genes and ADME (absorption, distribution, metabolism, and excretion) gene sets, found that patients with resistance to VPA had enrichment of rare genetic variants in the ADME gene set. No associations were found between the response to ASMs for the target genes in all three studied ASMs and ADME gene sets of LEV and LTG. The results suggest that resistance to ASMs may not be the consequence of a single or a few genetic variants, but rather determined by the cumulative effect of various genetic variants that eventually merge to alter the collective effect of drug responsiveness.

## Discussion

One of the weaknesses observed in these association studies is the lack of unified definitions of DRE and responsiveness. The ILAE introduced a new definition of DRE in 2010, and studies conducted before this may not have used this definition. It is anticipated that later association studies will adhere to a unified definition until any subsequent changes are made.

In addition to the varied definitions of DRE, the studies were also diverse in their approach. One approach focused on investigating the response to ASMs concerning the genetic variants of their mechanistic targets. Another approach involved examining the association between the response to various combinations of ASMs as a whole and the target of interest. Studies regarding variants in SCNs usually adopted the former approach, concentrating on ASMs that block SCNs, with a minority involving multiple ASMs. These studies could identify potential associations between genetic variants in SCNs and responses to SCN-blocking ASMs. Conversely, studies on calcium channel blockers, potassium channel openers, and GABA receptors typically included ASMs with diverse mechanisms. Consequently, their findings may only have revealed associations between genetic variants of the targets of interest and DRE, which may not necessarily disclose the association between the response to a particular ASM and its target. The identification of pharmacogenetic markers capable of predicting the response to an ASM prior to prescription would be more beneficial than predicting DRE to various ASMs. A more refined study approach that addresses the primary target and its corresponding ASMs is necessary before these findings can inform clinical practice in selecting the appropriate ASM. Given that SCN-blocking ASMs are the most frequently prescribed, studies on targets beyond SCNs may lack sufficient patient numbers to achieve statistical power. This could result in the inclusion of patients taking multiple ASMs for non-SCN blocker investigations. International collaboration in pharmacogenetic association studies could involve more participants and clarify the connection between ASM effectiveness and the genetic variants of their targets.

The aforementioned association studies primarily focused on common variants of the target genes, except for one study which examined rare variants of the glutamate receptor and their impact on response to PER (Lin et al., 2023). Common genetic variants are typically associated with quantitative phenotypes, with each locus contributing partially to various traits (Speliotes et al., 2010). Common variants often do not directly cause functional changes but rather indicate the presence of nearby functional variants due to linkage disequilibrium with observed phenotypes (Bodmer and Bonilla, 2008). In contrast, rare variants are more likely to have direct functional effects (Bodmer and Bonilla, 2008), although they are not as rare as deleterious mutations and have extremely low frequency in the population. These rare variants are evaluated based on computational algorithms suggesting potential functional consequences, particularly in genes relevant to the phenotypic pathophysiology (qualified variants) (Petrovski et al., 2013). Studies investigating the impact of rare variants on response to ASMs remain limited; however, they represent a promising avenue to augment our understanding of the functional consequences at a genetic level. These two types of variants are not mutually exclusive, and they have both been proposed to contribute to specific traits, with common variants modulating the effects of rare variants (Felix et al., 2006) and rare variants directly influencing trait development through functional consequences (Dickson et al., 2010).

Data regarding the functional consequences of these genetic variants are lacking. Therefore, the effect of the genetic variant and response to the drug cannot be determined. Variants found in studies of SNPs may not be the causal variants, since these common variants typically encompass a large number of variants in linkage disequilibrium, only some of which contribute to the phenotype (Weissbrod et al., 2020). For studies on rare variants, although the variants are predicted by computational algorithms, the true effects of these variants on protein structure and function are unknown (Livesey and Marsh, 2020). Understanding the consequences of these genetic variants can enhance our understanding of the reason for different drug responses. As previously discussed, the intronic variant SCN1A rs3812718 is associated with a reduced expression of the neonatal form of SCN1A, which is more sensitive to SCN blockers. This effect may be mitigated by escalating the dosage of SCN blockers (Thompson et al., 2011). The findings from genetic studies along with subsequent functional analyses can offer valuable insights into ASM prescription. This includes not only identifying the most effective medication but also anticipating individual responses to ASMs and adjusting treatment regimens accordingly.

Besides exploring the association of genetic variants and DRE, polygenetic risk score (PRS) offers an additional method for predicting the response to ASs. PRS hypothesizes that the genetic loading of a set of risk variants contributes to the development of a disease or particular trait with the need for little or no knowledge about the underlying mechanism (Lewis and Vassos, 2020). It is calculated using statistical algorithms and quantifies an individual’s genetic predisposition to a specific trait or disease by integrating data from multiple genetic markers across the genome. The PRS has been demonstrated to be a feasible method to predict the psychiatric side effects of LEV treatment for schizophrenia (Campbell et al., 2022). It may also be a reasonable approach to predict responsiveness, since many ASMs may have more than one mechanistic target, or in some cases an unknown mechanism.

By integrating genetic testing into clinical practice, personalized medicine facilitates the more precise selection and dosing of ASMs. This approach not only enhances the efficacy of ASMs but also minimizes the risk of ADRs, thereby improving patient outcomes and overall quality of life. With genetic testing-guided ASM selection, patients may achieve seizure freedom more rapidly. Moreover, this approach enables the identification of patients at risk of developing DRE earlier in their treatment journey, allowing for timely adjustments in treatment strategies or consideration of surgical options.

## Conclusion

This review analyzes pharmacogenetic studies, with a specific focus on the mechanistic targets of ASMs. Our findings show that it is currently challenging to definitively conclude that specific genetic variants in drug targets influence the response to ASMs. The existing pharmacogenetic studies often concentrate on a limited number of genetic variations, sometimes without a direct focus on the target of the studied ASMs, or involving a small number of participants. Studies exploring the association between DRE and the pharmacogenetics of ASMs are still in their infancy. Collaborative efforts among multiple centers are imperative to attain sufficient statistical power to draw conclusions regarding the relationship between the efficacy of ASMs and the genetic variants of their targets. In addition, tools such as PRS can be used to predict potential ADRs and integrate them into prediction methodologies. With further research into the pharmacogenetics of ASMs, it may soon be possible to predict the outcomes of specific ASMs by analyzing the genomic data of the patients, thereby eliminating the need for a trial-and-error approach.
